# Myopia in low-resource settings

**Published:** 2019-05-13

**Authors:** Ian G Morgan, Amanda Nicole French, Kathryn Rose

**Affiliations:** 1 Research School of Biological Science, Australian National University, Canberra, Australia.; 2 Senior Lecturer, Discipline of Orthoptics, Graduate School of Health, University of Technology, Sydney, Australia.; 3 Senior Lecturer, Discipline of Orthoptics, Graduate School of Health, University of Technology, Sydney, Australia.

**Increasing levels of myopia will pose particular challenges in low- or middleincome countries. Preventing or delaying myopia onset is crucial.**

Providing correction for myopia is a major challenge in low- and middle-income countries that have limited trained ophthalmic personnel and who are faced with other barriers; e.g., the cost of spectacles and misconceptions about spectacle wear. The challenge should not be underestimated: uncorrected refractive error is the single biggest cause of visual impairment and blindness worldwide.^1^

A second challenge is dealing with the pathological consequences of high myopia, defined as ≤ −5 dioptres (D). Myopia imposes a greater risk of retinal detachment from an early age,^2^ and signs of pathological myopia, such as myopic macular degeneration and staphyloma, can start to appear in young adults with high myopia and worsen with age. Myopic macular degeneration is the first or second cause of blindness already in East Asia.^3^ In addition, high myopia is the most common cause of choroidal neovascularisation in people under 50 years of age in high-income countries,^4^ and people with myopia are at greater risk of developing cataract and glaucoma.^5^ Many of these secondary conditions are clinically challenging.

As the prevalence of myopia has increased, early onset of myopia has become more common, allowing more time for progression to take place.^6^ Based on existing data, a child who first becomes myopic in mainland China at the age of 6.8 years is likely to develop high myopia. One who develops myopia at the age of 12.13 or later is unlikely to end up highly myopic. It is therefore very important to delay the onset of myopia and to control its progression.

Fortunately, we know how to do both. Increasing time spent outdoors during childhood delays the onset of myopia as demonstrated in randomised clinical trials,^7–9^ and as a system-wide programme in Taiwan. The aim should be to ensure that children in the primary school years get at least 2–3 hours a day outdoors, based on epidemiological evidence6 and clinical trial results.^7–9^

**Figure:**
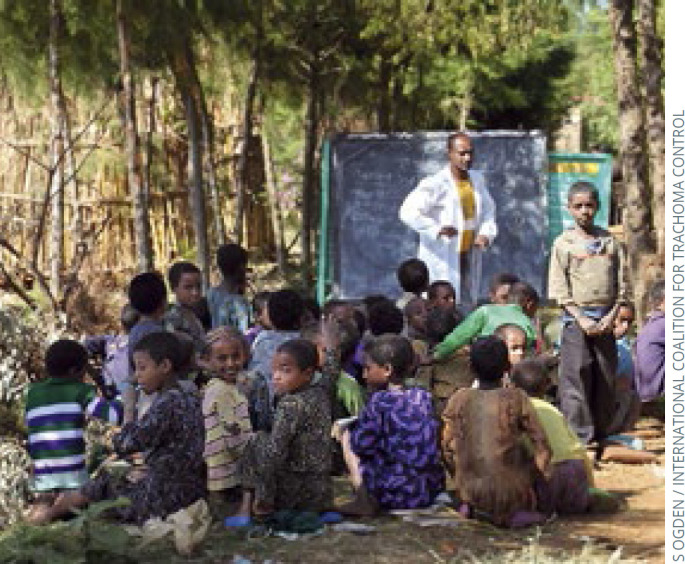
Learning outdoors for all or part of the day protects children against the onset of myopia. The light under trees is bright enough to make a difference. ETHIOPIA

There are several treatments that can control myopia by slowing down its progression.^10^ Daily low-dose atropine (p. 21) is reported to reduce progression by close to 60%, and executive bifocal spectacles with +1.5D near addition is reported to reduce progression by 50% (p. 19). In terms of cost, the use of low-dose atropine is likely to be preferred in low- or middleincome settings. These interventions, where available and affordable, could eliminate or drastically reduce the risk of developing high myopia/pathological myopia and the conditions associated with it (p. 5)

For progression control efforts to be effective, prompt detection and referral is essential (p. 15). Annual refraction and eye examination (p. 17) makes a lot of sense, at least until progression rates have slowed.

In countries already gripped by a myopia epidemic, putting these measures in place is urgent. In other countries, regular visual acuity screening for referral, as well as surveillance of trends, would also be useful. Increased time outdoors provides a simple, low-cost primary intervention that should be backed up with progression control, where possible. Where climate or pollution limits this possibility, innovative solutions such as bright classrooms, or bright desk lamps for home study, may be required.

We know how to control myopia – now we need to do it.
